# A Case of Hydrophthalmia Successfully Treated

**Published:** 1781-05

**Authors:** 


### SECTION II.

### Essays and Observations.

I. *A Case of Hydrophthalmia successfully treated. By Mr.* Edward Ford, *Surgeon to the Westminster General Dispensary. Communicated by* Samuel Foart Simmons, M. D. F. R. S. *Read May* 7, 1781.

MARY Bethell, aged twelve years, daughter of a publican in White-horse Yard, Drury-lane, lost the sight of her left eye by the small pox; and, in consequence of a blow received some time after, had a complete opacity of the cornea, attended with a dropsy of the aqueous humour.—It projected considerably, and was frequently subject to pain and inflammation, which often affected the sound eye.

It continued gradually to enlarge, till at length, in January 1776, it burst, and discharged a clear thin fluid; the tumour again filled, and in the month of June 1776, she was recommended as a patient to the Westminster General Dispensary. —I found the cornea of the left eye bulging out considerably from the orbit, so much indeed, that the eyelids could not be brought into contact tact with each other. The inconvenience of the disease was sufficient to justify an operation; it was a great deformity to the patient, and though the sight of the other eye was not then impaired, yet the frequent returns of inflammation to which it was liable, might have terminated in a total blindness.

Accordingly, with the concurrence of several gentlemen of the faculty, I made a large incision through the cornea, with the knife used in the extraction of the cataract; a tea-spoonful of clear water issued from the wound; but as in that operation we endeavour to obviate inflammation as much as possible; and in the present one my idea was to raise it in a moderate degree, I again introduced the knife, and made a small incision through which a portion of the vitreous humour escaped. Both eyes were then bound up with compresses dipt in saturnine water. The usual antiphlogistic regimen was pursued, and the symptoms of fever were not violent.

On the third day after the operation the eye was examined, but there was very little inflammation.

It is unnecessary to relate all the particulars of the treatment, I shall only observe, that in three weeks the disease returned.

It appeared to me that the relapfe was owing to a want of fufficient inflammation to bring on cohefion, I therefore prepared my patient for a few days, previous to a fecond operation, by diet and a gentle purge; and on Sunday the 8th of September, the found eye being bound up, and the difeafed one fixed by a fpeculum oculi, I paffed a large triangular needle, armed with a double thread of filk, through the anterior chamber of the eye. The thread being fecured, the faturnine water was applied, and the fame plan purfued as before, but with better fuccefs, for, within a fortnight, from the introduction of the feton, a confiderable inflammation with flight fuppuration had taken place; but it proceeded fo very gradually that the other eye had fuffered very little.

I now took out the feton, as the inflammation was ftill increafing, and it appeared to me to have arrived to fuch a crifis as would effect a cure. The inflammation foon fubfided, and the eye was reduced to rather lefs than its natural fize, in which ftate it has remained to this prefent time, being five years fince the operation.

We meet with little fatisfactory information concerning the hydrophthalmia in books of furgery.

gery. Porterfield mentions it, St. Yves likewife takes notice of this difeafe, but points out no mode of cure.—Nuck relates an account of his having cured a perfon labouring under this diforder, by making a fmall incifion in the cornea, to evacuate the aqueous humour, and afterwards bringing about a cohefion by preffure.—The fame method is likewife adopted by Heifter.—How far they mean to recommend this practice in thofe cafes where vifion is not totally obftructed by the hydrophthalmia, and the intent of the operator is to reftore or to improve fight, is not by any means the object of the prefent paper, its purport is to propofe a mode of cure in thofe cafes of hydrophthalmia that are attended with a complete opacity of the cornea.

*Golden-Square,* May 2, 1781.

